# Structural Analysis and Spatiotemporal Expression of *Atxn1* Genes in Zebrafish Embryos and Larvae

**DOI:** 10.3390/ijms222111348

**Published:** 2021-10-21

**Authors:** Franz Vauti, Viktoria Vögele, Isabel Deppe, Susanne T. Hahnenstein, Reinhard W. Köster

**Affiliations:** Division of Cellular & Molecular Neurobiology, Zoological Institute, Technische Universität Braunschweig, Spielmannstrasse 7, 38106 Braunschweig, Germany; v.voegele@tu-braunschweig.de (V.V.); isabeldeppe@arcor.de (I.D.); s.hahnenstein@posteo.de (S.T.H.); r.koester@tu-bs.de (R.W.K.)

**Keywords:** zebrafish, spinocerebellar ataxia, SCA, ataxin-1, *atxn1a*, *atxn1b*, *atxn1l*, phylogenetic tree, gene structure, gene expression, whole-mount in situ hybridization, WISH

## Abstract

Zebrafish have come into focus to model cerebellar diseases such as spinocerebellar ataxias (SCAs), which is caused by an expansion of translated CAG repeats in several unrelated genes. In spinocerebellar ataxia type 1 (SCA1), gain-of-function in the mutant ATXN1 contributes to SCA1’s neuropathy. Human ATXN1 and its paralog ATXN1L are chromatin-binding factors, act as transcriptional repressors, and have similar expression patterns. However, little is known about *atxn1* genes in zebrafish. Recently, two family members, *atxn1a* and *atxn1b*, were identified as duplicate orthologs of *ATXN1*, as was a*txn1l*, the ortholog of *ATXN1L*. In this study, we analyzed the phylogenetic relationship of the *atxn1* family members in zebrafish, compared their genetic structures, and verified the predicted transcripts by both RT-PCR and whole-mount in situ hybridization. All three genes, *atxn1a*, *atxn1b*, and *atxn1l*, show overlapping, but also distinct, expression domains during embryonic and larval development. While *atxn1a* and *atxn1l* display similar spatiotemporal embryonic expression, *atxn1b* expression is initiated during the onset of brain development and is predominantly expressed in the cerebellum throughout zebrafish development. These results provide new insights into *atxn1* genes and their expression patterns in zebrafish during embryonic and late-larval development and may contribute importantly to future experiments in disease modeling of SCAs.

## 1. Introduction

The autosomal dominant spinocerebellar ataxias (ADCAs) are a complex group of neurodegenerative diseases characterized by progressive dysfunction of the cerebellum and its afferent and efferent connections, which involve the brainstem and spinal cord [1,2]. ADCAs can be divided into seven different diseases of episodic ataxias (EA) with recurrent episodes of poor coordination and balance (ataxia) and spinocerebellar ataxias (SCAs), of which 43 different causative loci are known at the present time [3,4]. Seven of these different diseases (SCA1, SCA2, SCA3, SCA6, SCA7, SCA17, and DRPLA) are caused by CAG repeats in the coding exons of these unrelated genes, leading to polyglutamine expansions (poly-Q) in the translated gene products. The modified proteins are responsible for a predominant toxic gain-of-function, which depends on the length of the glutamine residues. There is an inverse correlation between the length of polyglutamine tracts and age and the appearance of neuropathological signs [5,6]. Although SCAs are genetically and clinically heterogeneous, the cerebellar pathologies are commonly associated with motor coordination failures of limbs and ataxic gait. Other clinical symptoms include variably pyramidal and extrapyramidal signs, peripheral neuropathy, pigmentary retinopathy, ophthalmoplegia, and dementia [7]. In contrast to the late-onset clinical features, the molecular and cellular events that underlie the neurological symptoms are still poorly understood.

To gain insights into the mechanisms that contribute to ataxic phenotypes, the zebrafish *Danio rerio* has emerged as a useful model system to study brain development, neuronal function, network activity, and neuropathy in vertebrates. Zebrafish models are gaining popularity in neurobiology due to their optic transparency, the similar general organization of their CNS to other vertebrates, and the neuronal genes, which show reminiscent functions in humans [8]. These conserved genetics allow both the elucidation of normal gene function in vertebrates and the analysis of genetic dysfunction and the mechanisms involved in inherited neurodegenerative diseases. Neuropathological processes with phenotypic overlaps give insights into common molecular pathways that are responsible for similar behavioral patterns between species. A wide spectrum of behavioral protocols have been utilized to support behavioral phenotype analysis in regards to compound screens for the therapeutic treatment of the CNS in zebrafish embryos, larvae, and adults [9,10,11,12,13,14]. Therefore, zebrafish models have many advantages and have become increasingly important to the study of neurodegenerative diseases such as SCAs, which are incurable, debilitating disorders.

There is still a lack of adult zebrafish SCA models and a huge gap in the knowledge of *ataxin* genes and their functions in zebrafish. Therefore, it is important to expand the range of zebrafish models for the study of the different types of dominant ataxias [4]. One of the frequent dominant ataxias in which a similar zebrafish protein function is expected is spinocerebellar ataxia type 1 (SCA1). SCA1 is characterized by cerebellar and brainstem degeneration [5]. The fatal late-onset disorder is caused by an expansion of CAG repeats in the coding region of the *ATXN1* gene. The accumulation of polyglutamine-expanded ATXN1-poly-Q results in selective neuronal degeneration in the cerebellum and brainstem, causing the loss of motor coordination, which induces swallowing and breathing difficulties of patients and leads to premature death [15]. Although ATXN1 is broadly expressed in the human brain, the neuronal degeneration caused by *poly-Q-ATXN1* selectively affects the neurons of the cerebellum and the brainstem [5,6,15].

In a previous work, three *atxn1* gene family members were identified in zebrafish: two *ATXN1* orthologs, *atxn1a* and *atxn1b*, and the *ATXN1L* ortholog *atxn1l* [16]. The authors demonstrated that all are expressed in the developing cerebellum and in adult Purkinje cells by pan-ATXN1 immunohistochemistry. This suggests that the zebrafish is a suitable model system with which to study the SCA1 pathogenesis. However, for further analysis of the distinct *atxn1* genes in zebrafish, a broader knowledge of their individual spatiotemporal expression is missing and may be beneficial for future research. Here, we compare the genetic structures of the *atxn1* genes in the zebrafish genome and analyze the phylogenetic relationship of their deduced proteins in vertebrates. We prove the bioinformatically predicted transcript variants of *atxn1a*, *atxn1b,* and *atxn1l* genes and provide new insights into shared and different expression domains during zebrafish development.

## 2. Results

### 2.1. ATXN1 Family Proteins Are Phylogenetically Conserved in Vertebrates

**Figure 1:**
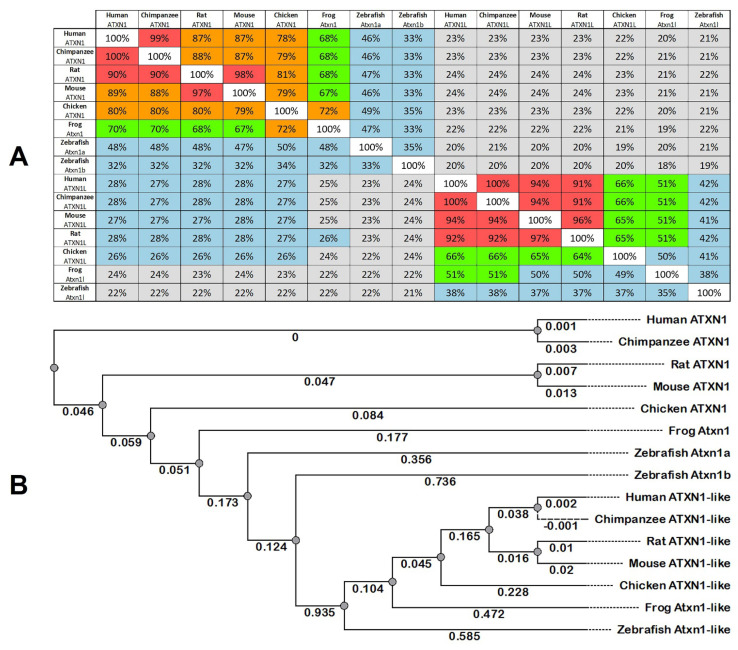
ATXN1 and ATXN1L proteins are highly conserved in vertebrates. (**A**) Multiple sequence alignment distance matrix indicated for Ataxin-1 proteins (*Homo sapiens*: NP_001121636.1; *Pan troglodytes*: XP_001180180.2; *Rattus norvegicus*: XP_038951298.1; *Mus musculus*: NP_001186234.1; *Gallus gallus*: XP_003640803.1; *Xenopus tropicalis*: XP_002932838.1; *Danio rerio*: NP_001038291.1; *Danio rerio*: XP_005158216.1) and Ataxin-1-like proteins (*Homo sapiens*: NP_001131148.1; *Pan troglodytes* XP_016885624.1; *Mus musculus*: NP_001084399.1; *Rattus norvegicus*: XP_008880885.2; *Gallus gallus*: XP_015148186.1; *Xenopus tropicalis*: XP_002938622.1; *Danio rerio* XP_001333881.2). (**B**) Phylogenetic tree of Ataxin-1 and Ataxin-1-like amino acid sequences was inferred by the PHYLIP neighbor-joining method (Unipro UGENE software analysis tool) and the Jones–Taylor–Thornton distance matrix model.

Disease-causing genes in humans inform the role of animal models used to study the function and pathogenesis of genetic disorders. Similarities of protein structures and functions between species are a prerequisite for the use of model systems to unravel normal or pathological cellular processes. We used the online databases from Ensembl and the National Center for Biotechnology Information (NCBI) (Table S1) to analyze the similarities of human ATXN1 (NP_001121636.1) and human ATXN1L (NP_001131147.1) protein sequences to their corresponding orthologs in zebrafish and other vertebrates (Figure 1A). The human ATXN1 (815 amino acid residues, aa) shared many similarities to the mammalian orthologs (specifically, chimpanzee 99%, rat 87%, and mouse 87%) and less to the other vertebrates (specifically, chicken 78% and frog 68%). The teleost zebrafish shared 46% and 33% identity in their Atxn1a (781 aa) and Atxn1b (827 aa), respectively, to their human orthologs. The zebrafish Atxn1a protein indicated a 50% similarity to the ortholog found in chickens; 48% in humans, chimpanzees, rats, and frogs; 47% in mice; and 35% to the atxn1b paralog in its own species.

Compared to ATXN1 (815 aa), the human ATXN1L protein is shorter (689 aa), and the sequence comparison revealed a 23% similarity between both proteins. The comparison of the human ATXN1L protein to the vertebrate orthologs revealed a high identity to mammalian proteins (chimpanzee 100%, mouse 94%, and rat 91%) and less to those found in chickens (66%) and frogs (51%). The zebrafish Atxn1l protein (765 aa) had the longest aa chain of the selected vertebrate orthologs and was 42% identical to the human protein. The zebrafish Atxn1l protein was most similar to human and chimpanzee proteins (38%), but also to those of rats, mice, chickens (37% each), and frogs (35%) (Figure 1A).

The ATXN1 and ATXN1L family members share conserved protein domains between species. A ClustalW alignment of the vertebrate proteins revealed regions of high homology (Figure S1). The N-terminal (Figure S1A) and the C-terminal (Figure S1E) ends of the proteins, as well as the AXH domain (Figure S1C,D), represent three highly conserved homologous regions found in all proteins, whereas the polyglutamine tract in the ATXN1 was specific only to the hominoid lineage of human and chimpanzee (Figure S1B). In addition, all ATXN1 family members contained a nuclear localization signal (nls: RKRRWSAPE), which was not the case for the ATXN1L (Figure S1D).

Following the sequence comparison of the ATXN1 and ATXN1L proteins and their orthologs, we performed a phylogenetic analysis of the proteins to unravel the relationships of zebrafish Atxn1a and Atxn1b proteins to each other and to their human orthologs as well as to those of the other vertebrates (Figure 1B). The phylogenetic tree was constructed using the Unipro UGENE software analysis tool, according to the same amino acid sequences of the indicated species (Table S1). Our phylogenetic analysis indicated two clusters of ATXN1 proteins and ATXN1L proteins that were evolutionarily more distant than each of the two proteins in the different vertebrates. The ATXN1 clades showed a close relationship in hominids (human and chimpanzee) and rodents (rats and mice) but less so in chickens, frogs, and zebrafish. The same distant relationships were obvious for the ATXN1L cluster between the different classes. The overall phylogenetic analysis showed that the zebrafish Atxn1b protein was the evolutionarily most distant protein to the human ATXN1 protein. Interestingly, the zebrafish Atxn1b showed the evolutionarily closest relationship to its own Atxn1l protein and to that of the other selected vertebrates.

### 2.2. Structure and Transcript Variants of Atxn1 Genes in Zebrafish

**Figure 2:**
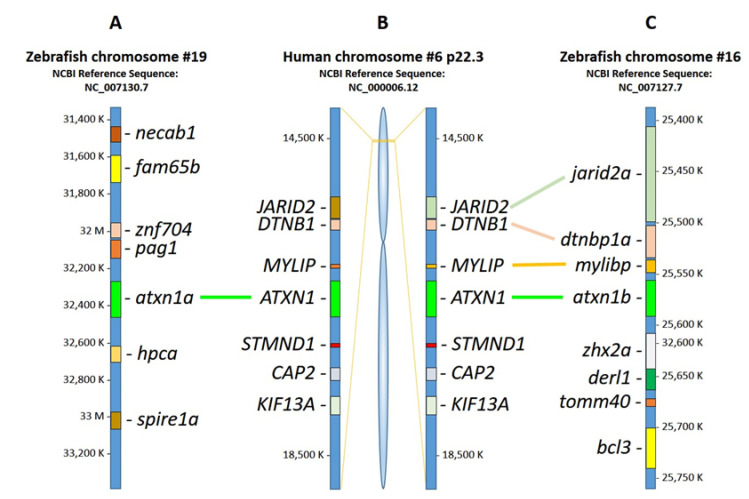
Synteny analysis of human *ATXN1* and zebrafish *atxn1a* and *atxn1b*. Schematic diagram showing the results of NCBI and Ensembl database analysis. (**A**) Zebrafish *atxn1a* is located on chromosome 19 (plus strand), (**B**) human *ATXN1* gene on human chromosome 6p22.3 (minus strand), and (**C**) zebrafish *atxn1b* on chromosome 16 (minus strand). Comparative synteny analysis of the human *ATXN1* locus shows no synteny to *atxn1a* genomic regions in zebrafish. In contrast, a flanking region to human *ATXN1* encompassing the genomic loci of *MYLIP*, *DTNB1*, and *JARID2* shows conserved synteny to genomic regions of *atxn1b*, *mylbp*, d*tnbp1a,* and *jarid2a* in zebrafish.

According to the NCBI gene database, the human *ATXN1* gene is located on chromosome 6p22.3 (minus strand) with 11 exons. In contrast to mammals, whole-genome duplication occurred in the teleost lineage about 320 million years ago [17]. In zebrafish, two paralogs of the *ATXN1* gene exist: *atxn1a* on chromosome 19 (plus strand) and *atxn1b* on chromosome 16 (minus strand). We performed a comparative synteny analysis of the human and zebrafish gene loci (Figure 2). At the genomic level, *atxn1a* showed no synteny (Figure 2A) to the flanking chromosomal regions of *ATXN1* (Figure 2B). In contrast, for the zebrafish genes *jarid2*, *dtnbp1a*, *mylibp*, and *atxn1b*, we identified conserved synteny to the loci of *JARID2*, *DTNB1*, *MYLIP*, and *ATXN1* in humans. However, synteny was shown only for one of the two flanking regions of *ATXN1*, whereas the genes of the second region (*STMND1*, *CAP2*, and *KIF13A*) around the *atxn1b* locus were absent. In summary, *atxn1a* on chromosome 19 showed no synteny to the human *ATXN1* locus on chromosome 6p22.3 while *atxn1b* on chromosome 16 displayed partially conserved synteny.

#### 2.2.1. *Atxn1a* Gene in Zebrafish Encodes Four Transcript Variants

*Atxn1a* is found on chromosome 19 (NC_007130.7: position 32266582–32464396) with eight exons. Based on the genomic data of the *atxn1a* gene in zebrafish, one coding sequence of *atxn1a* mRNA (NM_001044826.1, length: 2346 bp) is annotated in the NCBI database. Four additional deduced *atxn1a* transcript variants are predicted by bioinformatic software analysis. The four transcript variants are: X1 mRNA (XM_017352239.2, length: 5928 bp); X2 mRNA (XM_009294318.3, length: 5768 bp), X3 mRNA (XM_005159620.4, length: 5866 bp), and X4 mRNA (XM_005159619.4, length: 5654 bp).

**Figure 3:**
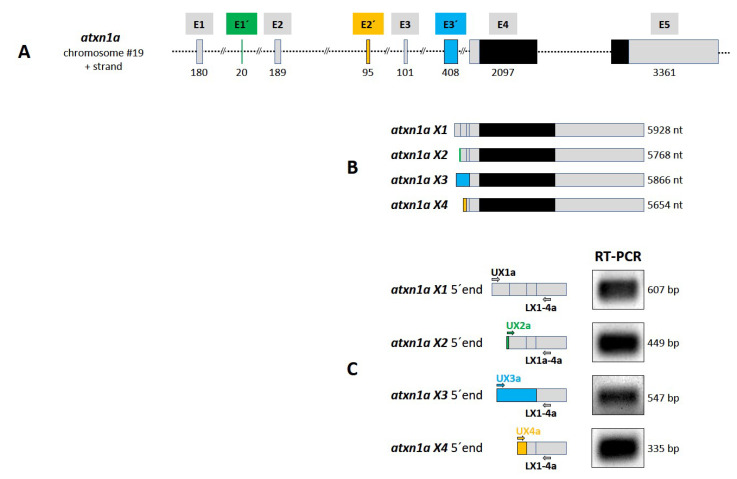
*Atxn1a* gene in zebrafish. (**A**) Schematic presentation of the structure of *atxn1a* gene on chromosome 19 plus strand. The gene consists of eight exons (E1, E1′, E2, E2′, E3, E3′, E4, and E5) containing the coding sequence (black boxes) in the last two exons E4 and E5. (**B**) Four different transcripts (X1–X4) are predicted in the NCBI database. The *atxn1a* X1 variant is transcribed from five exons (E1, E2, E3, E4 and E5; grey boxes) such as the X2 variant with the alternative first exon E1′ (green box). The X3 variant contains the alternative exon E3′ (blue box) first, followed by E4 and E5. The X4 transcript variant is encoded by the alternative exon E2′ (orange), followed by sequences from exons E3, E4, and E5. (**C**) Experimental verification of predicted *atxn1a* X1–X4 variants. Four different upper primers (UX1a, UX2a, Ux3a, and Ux4a), specific for the alternative exons (shown in A and B), in combination with a lower primer (LX1-4a), specific for exon E4 sequence, were designed for RT-PCR of zebrafish cDNA as template to amplify the 5′ end of each variant (left panel). The lengths of the amplicons (right panel) and the following sequencing confirmed the predicted *atxn1a* X1–X4 transcript variants.

Using the sequence data, we performed a BLAST analysis of the predicted *atxn1a* transcripts in the genome database in order to identify the genomic structure of the *atxn1a* gene in the zebrafish genome (Table S2). The X1 transcript variant of the *atxn1a* gene was encoded by five exons spanning a genomic region of 197,815 bp (Table S2A). All X1–X4 transcript variants differed in their first exon (X1: E1; X2: E1′; X3: E3’and X4: E2′). Compared to X1, variant X2 shared exons E2, E3, E4, and E5; variant X3 shared exons E4 and E5; and variant X4 shared exons E3, E4, and E5. Together, the four predicted transcript variants differed in exon usage in the 5′untranslated region (5′ UTR). The distribution of the eight different exons of the *atxn1a* gene is schematically illustrated in Figure 3A. The coding sequence of the open reading frame is indicated (black boxes) in the larger exons E4 and E5. In order to verify the predicted *atxn1a* X1–X4 transcript variants (Figure 3B) in zebrafish, we performed an RT-PCR analysis. We isolated the total RNA from adult zebrafish for cDNA synthesis and used transcript specific primer pairs, forward primers (UX1a, UX2a, UX3a, UX4a), and one for all specific lower primers, LX1-4a (Table S3), for the specific transcript amplifications in the PCR (Figure 3C). The PCR amplicons (Figure 3C, right panel) showed the expected fragment sizes of the four different X1–X4 transcripts. Purification of the fragments, which was followed by sequencing, confirmed all four transcript variants to be expressed in zebrafish.

#### 2.2.2. *Atxn1b* Gene in Zebrafish Encodes Two Transcript Variants

**Figure 4:**
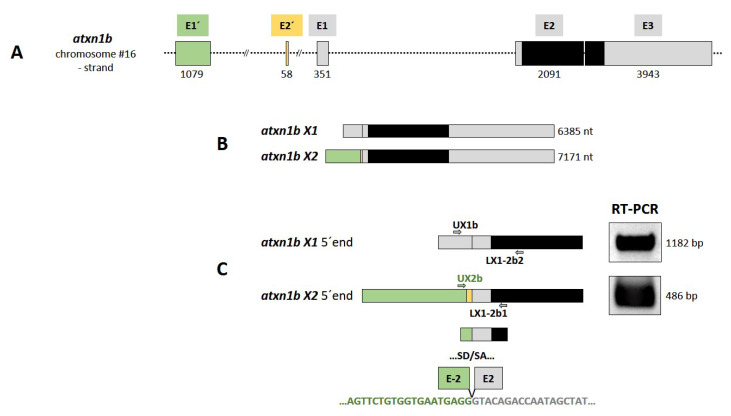
*Atxn1b* gene in zebrafish. (**A**) Schematic presentation of the structure of *atxn1b* gene on chromosome 16 minus strand. The gene consists of five exons (E1′, E2′, E1, E2, and E3) containing the coding sequence (black box) in the last two exons E2 and E3. (**B**) Two different transcripts (X1 and X2) are predicted in the NCBI database. The *atxn1b* X1 variant is transcribed from three exons (E1, E2, and E3; grey boxes). The X2 variant contains two alternative exons: E1′ (green box) and E2′ (yellow box) upstream of E1. (**C**) Experimental verification of predicted *atxn1b* X1 and X2 variants. Two different primer pairs (UX1b/LX1-2b2 and Ux2b/LX1-2b1) specific for the alternative 5′ exons (shown in A and B) were designed for RT-PCR of zebrafish cDNA templates to amplify the 5′ ends of each variant (left panel). The lengths of the amplicons (right panel) and their sequencing confirmed the predicted *atxn1b* X1 and X2 (E1′) transcript variants. However, the X2 variant lacks the E2′ sequence (lower panel).

*Atxn1b* is located on chromosome 16 (NC_007127.7: position 25557382–25593182) and contains five exons. Two transcript variants, X1 and X2, are predicted for the *atxn1b* gene: variant X1 mRNA (XM_005158159.4, length: 6385 bp) and variant X2 mRNA (XM_689102.6, length: 7171 bp) (Table S2B). The X1 variant contains three exons (E1, E2, E3) in a 10,892 bp genomic region. Similar to the *atxn1a* gene locus, the 5′-exon usage differs also in the *atxn1b* gene locus. The predicted X2 transcript variant contains two different exon sequences in the 5′ UTR. The X2 variant is encoded by four exons (E1′, E2′, E2, and E3) in a larger genomic region with a size of 35,801 bp because exons E1′ and E2′ are located upstream of E1. The distribution of the five different exons of the *atxn1b* gene is schematically illustrated in Figure 4A, along with the coding region and open reading frames in exons E2 and E3. The predicted *atxn1b* X1 and X2 transcript variants (Figure 4B) have been confirmed by the RT-PCR analysis (Figure 4C) as transcripts in adult zebrafish. Specific primer combinations (UX1b/LX1-2b2 and Ux2b/LX1-2b1, Table S3) amplified both transcripts with expected fragment sizes (Figure 4C, right panel). Purification of the fragments and the following sequencing confirmed both predicted transcripts in adult zebrafish tissues. However, the exon E2′ sequence was missing, indicating that it was either not expressed or was spliced out in the X2 transcript.

#### 2.2.3. *Atxn1l* Gene in Zebrafish Encodes One Transcript Variant

**Figure 5:**
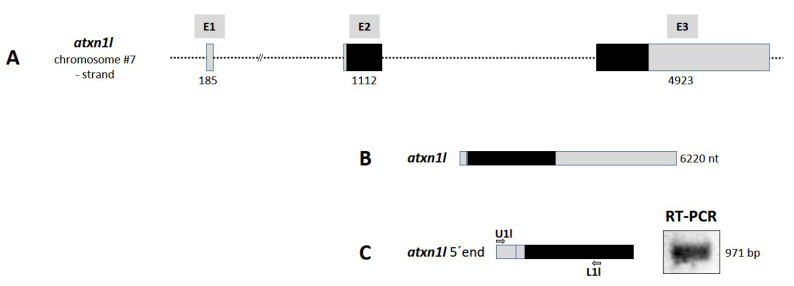
*Atxn1l* gene in zebrafish. (**A**) Schematic presentation of the structure of *atxn1l* gene on chromosome 7 minus strand. The gene consists of three exons (E1, E2, and E3) containing the coding sequence (black box) in the exons E2 and E3. (**B**) Only one *atxn1l* transcript is predicted from three exons (E1, E2, and E3; grey boxes). (**C**) Experimental verification of the predicted *atxn1l* transcript. The upper primer (U1l), specific for exon E1 sequence, in combination with the lower primer (L1l), specific for exon E3 sequence, were designed for RT-PCR of zebrafish cDNA template to amplify the 5′ end of the transcript (left panel). The length of the amplicon (right panel) and the following sequencing confirmed the predicted *atxn1l* transcript variant.

The human *ATXN1L* gene contains three exons and is located on chromosome 16q22. The zebrafish gene locus of the paralog *atxn1l* is found on chromosome 7 (NC_007118.7: position 69.411.766–69.429.487) with three exons in a region of 17,722 bp (Table S2C). In contrast to *atxn1*, no duplicate *atxn1l* gene was found in the zebrafish genome. The schematic drawing of the *atxn1l* gene locus is illustrated in Figure 5A. Only one *atxn1l* transcript is predicted in the database (XM_001333745.7, length: 6220 bp) (Figure 5B). We could experimentally confirm this transcript by RT-PCR analysis with the *atxn1l* specific primer pair U1l/L1l (Table S3) and using cDNA synthesized from adult zebrafish total RNA. The expected PCR product of 970 bp (Figure 5C, right panel) was purified and sequenced as evidence.

### 2.3. Expression Pattern of Atxn1 Genes during Zebrafish Development

**Figure 6:**
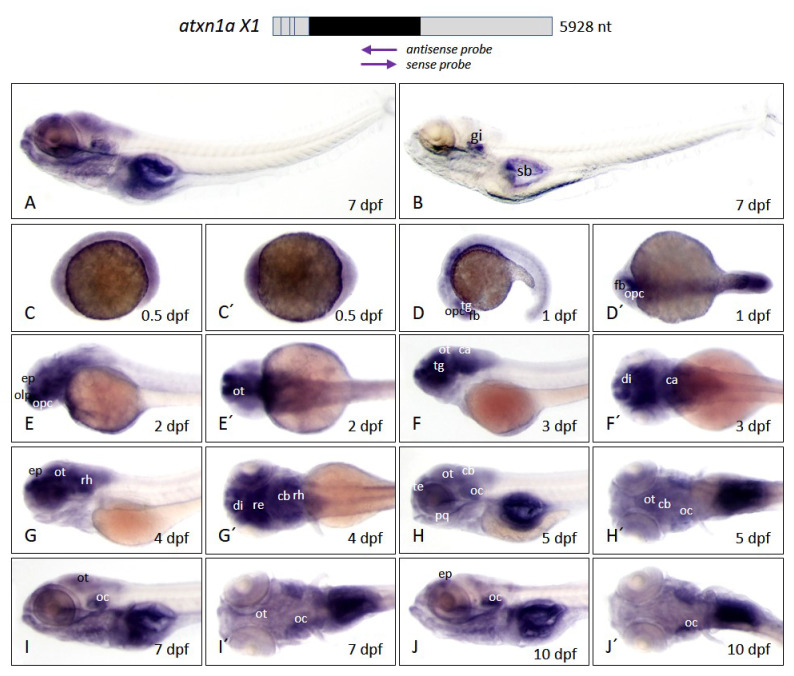
*Atxn1a* expression pattern in zebrafish embryos and larvae. Whole-mount in situ hybridization was performed in embryos and larvae of the *brass* line at different developmental stages. (**A**) Expression domains of *atxn1a* in whole larva (7 dpf) detected by antisense probes in the coding region of the transcript, indicated by the left arrow below the schematized transcript (upper panel). (**B**) Sense probes (right arrow, upper panel) indicate background staining in whole larva (7 dpf). Images (**C**–**J**) show lateral views and (**C′**–**J′**) dorsal views of *atxn1a* expression domains during embryonic and larval development (0.5, 1, 2, 3, 4, 5, 8, and 10 dpf). Abbreviations: ca (cerebellar anlage), cb (cerebellum), di (diencephalon), ep (epiphysis), fb (forebrain), gi (gills), oc (otic capsule), olp (olfactory pit), opc (optic capsule), ot (optic tectum), pq (palatoquadrate), re (retina), rh (rhombencephalon), sb (swim bladder), te (telencephalon), tg (tegmentum).

**Figure 7:**
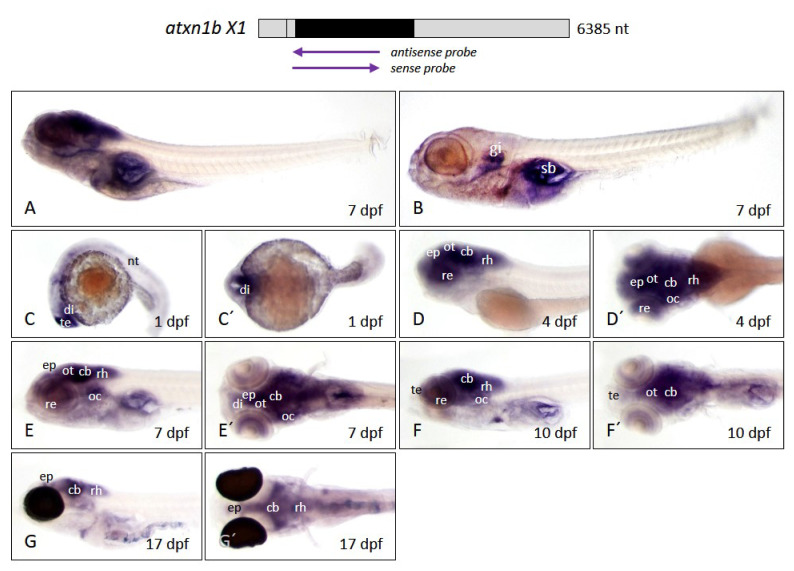
*Atxn1b* expression pattern in zebrafish embryos and larvae. Whole-mount in situ hybridization was performed in embryos and larvae of the *brass* and *casper* line at different developmental stages. (**A**) Expression domains of *atxn1b* in whole larva (7 dpf) detected by antisense probes in the coding region of the transcript, marked by the upper left arrow below the schematized transcript (upper panel). (**B**) Background staining in whole larva (7 dpf) after use of sense probes (right arrow, upper panel). Lateral (**C**–**F**) and dorsal (**C′**–**F′**) views of *atxn1b* expression domains of brass zebrafish during embryonic and larval development (1, 4, 8, and 10 dpf). (**G**) lateral and (**G′**) dorsal view of *atxn1b* expression domains in 17 dpf *casper* larva. Abbreviations: cb (cerebellum), di (diencephalon), ep (epiphysis), gi (gills), nt (neural tube), oc (otic capsule), ot (optic tectum), re (retina), rh (rhombencephalon), sb (swim bladder), te (telencephalon).

**Figure 8:**
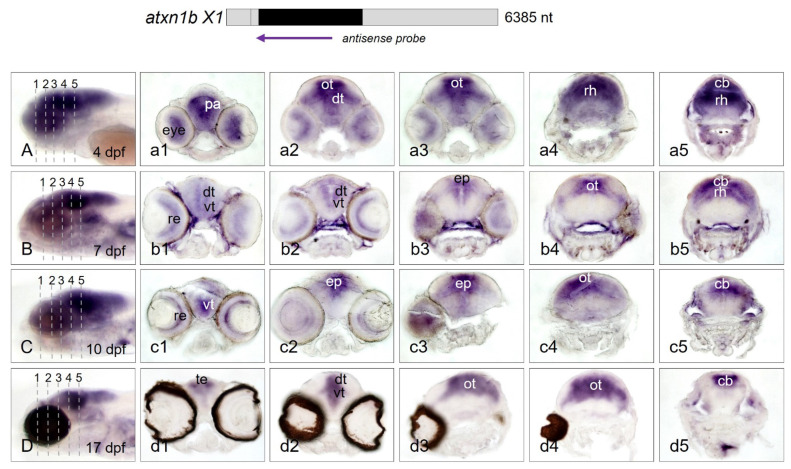
*Atxn1b* expression domains in cryosectioned zebrafish larvae. Whole-mount in situ hybridization was performed with larvae of the *brass* and *casper* line at different developmental stages. Expression domains of *atxn1b* in whole larva (7 dpf) were detected by antisense probes for the coding region of the transcript (indicated in the upper panel). (**A**–**D**) show transverse cryosections (7 µm) of five different regions of the head (left panels). Serial rostral to caudal views of the head region are shown for *brass* larvae at developmental stages of 4 dpf (**a1**–**a5**), 7 dpf (**b1**–**b5**), 10 dpf (**c1**–**c5**), and for the *casper* larva at 17 dpf (**d1**–**d5**). Abbreviations: cb (cerebellum), dt (dorsal thalamus), ep (epiphysis), ot (optic tectum), pa (pallium), re (retina), rh (rhombencephalon), te (telencephalon), vt (ventral thalamus).

**Figure 9:**
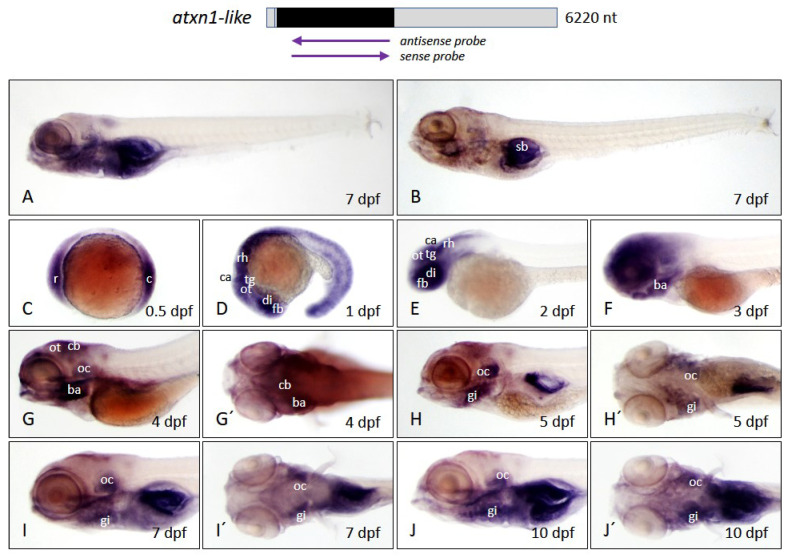
*Atxn1l* expression pattern in zebrafish embryos and larvae. Whole-mount in situ hybridization was performed in embryos and larvae of the *brass* line at different developmental stages. (**A**) Expression domains of *atxn1l* in whole larva (7 dpf) detected by antisense probes in the coding region of the transcript, marked by the upper left arrow below the schematized transcript (upper panel). (**B**) Background staining in whole larva (7 dpf) after use of sense probes, (right arrow, upper panel). (**C**–**J**) lateral and (**G′**–**J′**) dorsal views of *atxn1l* expression domains during embryonic and larval development (0.5, 1, 2, 3, 4, 5, 8, and 10 dpf). Abbreviations: ba (branchial arches), c (caudal), ca (cerebellar anlage), cb (cerebellum), di (diencephalon), fb (forebrain), gi (gills), oc (otic capsule), ot (optic tectum), r (rostral), rh (rhombencephalon), sb (swim bladder), tg (tegmentum).

In a recent work, the expression of *atxn1a* and *atxn1b* has been shown in the cerebellar anlage of 28 hpf zebrafish embryos [16]. Here, we determined the expression pattern of the three *atxn1* homologs during zebrafish embryonic and larval development. Using specific antisense probes for each of the three transcripts (Table S4), whole-mount in situ hybridization was performed in 0.5–5 dpf embryos and 7–10 dpf zebrafish larvae of the *brass* line (Figure 6, Figure 7, Figure 8 and Figure 9). Expression patterns are displayed as lateral and dorsal views. The specificity of the antisense probes for *atxn1a*, *atxn1b,* and *atxn1l* transcripts was initially tested in 7 dpf whole-mount larvae. The sense probes served as controls for the background staining.

#### 2.3.1. Expression of *Atxn1a* Is Enhanced during Embryonic Brain Development and Reduced at Larval Stages

*Atxn1a* was mainly expressed in the head region of the larval body (Figure 6A). In comparison, the sense probe control showed some background staining in the swim bladder and gills (Figure 6B). A weakly distributed expression of *atxn1a* was evident in the whole embryo during the segmentation phase at the 0.5 dpf developmental stage (Figure 6C,C′), with increasing signals in the forebrain at 1 dpf (Figure 6D,D′). The tegmentum and areas of the optic capsule showed stronger expression than the remaining part of the embryo. A very pronounced expression domain was seen in the olfactory pit, the epiphysis, and the optic tectum at 2 dpf (Figure 6E,E′), extending to the tegmentum, the optic tectum, the diencephalon, and the cerebellar anlage at 3 dpf (Figure 6F,F′). In addition, high levels of *atxn1a* transcripts were detected in the epiphysis, the optic tectum, the diencephalon, the retina, the cerebellar anlage, and rhombencephalon at 4 dpf (Figure 6G,G′), and in the telencephalon, the optic tectum, and otic capsules at 5 dpf (Figure 6H,H′). The expression was particularly pronounced in the palatoquadrate, which became evident later in development. In the cerebellum, a more diffuse, weaker expression of *atxn1a* was detectable, though not as distinct as in other brain areas. The signal in the optic tectum was clearly seen up to a developmental stage of 5 dpf. In the following stages, *atxn1a* expression decreased at 7 dpf and 10 dpf (Figure 6I,I′,J,J′). The intense signals in the optic tectum also disappeared with advancing age. Only the otic capsule maintained a higher level of *atxn1a* expression. Together, the temporal variation of the *atxn1a* expression in the brain regions of the zebrafish was evidently dependent on the different developmental stages of the zebrafish.

#### 2.3.2. Expression of *Atxn1b* Is Pronounced in Midbrain/Hindbrain Regions at Larval Stages

*Atxn1b* expression was detected almost exclusively in the zebrafish larval brain using an antisense probe (Figure 7A). The sense probe generated background staining in the gills and the swim bladder (Figure 7B). The expression domain of *atxn1b* was predominantly present in the telencephalon and basal diencephalon of the developing zebrafish embryo at 1 dpf (Figure 7C,C′) and less in the neural tube. No transcripts were detectable in the posterior regions from 4 dpf onward (Figure 7D,D′). However, there was a strong expression of *atxn1b*, especially in areas such as the epiphysis, the retina, the optic tectum, the cerebellum, the otic capsule, and the rhombencephalon. This pattern of expression was found again at 7 dpf (Figure 7E,E′), although it was less distributed. A clear expression in the retina of the eye was seen as a ring around the lens. The expression in the diencephalon persisted as well as in the optic tectum, the cerebellum, the otic vesicles, and the rhombencephalon. The *atxn1b* transcripts disappeared in the forebrain as the embryo aged, and weak or no expression was detectable in the telencephalon at 10 dpf (Figure 7F,F′). The site of *atxn1b* expression was still in the retina around the lens as well as the optic tectum, the cerebellum, the otic capsules, and the rhombencephalon. In contrast to the *atxn1a* gene, *atxn1b* was clearly expressed in the hindbrain of 10 dpf zebrafish larvae. We performed whole-mount in situ hybridization in transparent *casper* larvae (Figure 7G,G′) to verify that the expression domain in the cerebellum was detectable even at older stages. A strong expression of *atxn1b* was obvious in this region in 17 dpf larvae. Because *casper* larvae have pigmented eyes, the staining in the area of the eye could not be considered. Together, the range of *atxn1b* expression in the forebrain decreased with increasing embryonic age until the expression was almost exclusively concentrated in the midbrain and hindbrain regions.

To support these findings, cross-sections of stained embryos and larvae were prepared (Figure 8A–D) to allow for microscopic observation of the cell layers. Five regions (#1–5) of the heads from anterior to posterior regions are illustrated as transverse sections. *Atxn1b* was expressed in the forebrain region of the brain, in the eye, and pallium (Figure 8(a1)). This was followed by the optic tectum and dorsal thalamus (Figure 8(a2,a3)) as a distinct site of expression. Subsequently, the rhombencephalon and the established cerebellum were identified as an expression domain of *atxn1b* (Figure 8(a4,a5)). Similar areas of expression were seen in the 7 dpf larvae (Figure 8(b1–b5)). However, a limited range of expression was obvious. Expression domains were determined to be the retina of the eyes, the dorsal and ventral thalamus, and the pineal gland (Figure 8(b1–b3)) as well as the optic tectum (Figure 8(b4)) and the cerebellum (Figure 8(b5)). The expression pattern of *atxn1b* in 10 dpf larvae (Figure 8(c1–c5)) was similar to that of the developmental stage at 7 dpf. The section showed staining in the retina and ventral thalamus (Figure 8(c1)), the epiphysis (Figure 8(c2,c3)), and the optic tectum (Figure 8(c4)). *Atxn1b* was expressed in the area posterior to the eyes of the zebrafish embryo in the cerebellum, as this area was highlighted by staining (Figure 8(c5)). The expression domains of *atxn1b* were more constricted but still evident in 17 dpf zebrafish larvae (Figure 8(d1–d5)). The staining here was limited to individual areas rather than the entire brain area. The olfactory bulbs (Figure 8(d1)), the dorsal and ventral thalamus (Figure 8(d2)), and the optic tectum (Figure 8(d3,d4)) were determined as the site of expression. Remarkably, the cerebellum was the prominent expression domain of *atxn1b* in 17 dpf larvae (Figure 8(d5)). In summary, the results of the WISH analysis showed that *atxn1b* likely plays an important role in zebrafish neuronal development and differentiation. Individual brain areas where the *atxn1b* expression was maintained or enhanced during development have been identified, such as the telencephalon, optic tectum, and cerebellum.

#### 2.3.3. Expression of *Atxn1l* Is Increased in the Early Embryonic Brain and Decreases in the Larval Stage

*Atxn1l* transcripts were mainly detected by the antisense probe in the ventral head region (Figure 9A) of the larval zebrafish body while the sense probe resulted in background staining of the swim bladder (Figure 9B). *Atxn1l* showed a stronger expression in rostral and caudal segments of the zebrafish embryo at 0.5 dpf (Figure 9C), which shifted to the anterior part at 1 dpf (Figure 9D) and 2 dpf. The highest expression was indicated in the developing CNS including the forebrain, the diencephalon, the optic tectum, the tegmentum, the cerebellar anlage, and the rhombencephalon at 2 dpf (Figure 9E). An additional expression domain was found in the branchial arches at 3 dpf (Figure 9F). *Atxn1l* was downregulated caudally to the head region from stages 3 dpf to 10 dpf. A decline of expression was also detectable in most brain regions, starting at 4 dpf (Figure 9G,G′), when transcripts were still present in the optic tectum and the cerebellum, but primarily in the otic capsules and the branchial arches. The expression pattern of *atxn1l* was reduced in almost all brain regions and increased in restricted domains of the otic capsules and gills at the later stages of 5, 8, and 10 dpf (Figure 9H,H′,I,I′,J,J′).

### 2.4. Expression Levels of Atxn1 Genes during Zebrafish Development

**Figure 10:**
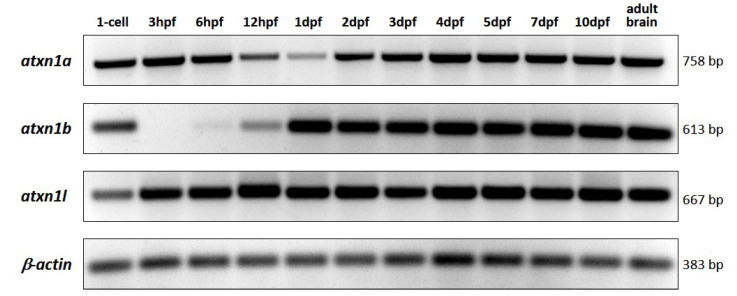
RT-PCR analysis of *atxn1* gene expression level in whole-body zebrafish. Total RNA was isolated from embryos and larvae at different developmental stages (single-cell, 3 hpf, 6 hpf, 12 hpf, 1 dpf, 2 dpf, 3 dpf, 4 dpf, 5 dpf, 7 dpf, and 10 dpf) and from adult zebrafish brains. Gene-specific primer pairs for *atxn1a*, *atxn1b,* and *atxn1l* were used to amplify the reverse transcribed cDNAs. The primer pair for *β-actin* was used as a control. The sizes of the amplicons are given.

In addition to analysing the spatial expression of *atxn1* genes in zebrafish embryonic and larval tissues, we examined the transcript levels of *atxn1a*, *atxn1b*, and *atxn1l* by RT-PCR in whole embryos, larvae, and adult brains. Total RNA was isolated and reverse transcribed. cDNAs were amplified by PCR with gene-specific *atxn1* and *β-actin* primer pairs (Table S5), and amplicons were analysed after gel electrophoresis (Figure 10). *β-actin* was equally expressed at all developmental stages and in the adult brain and served as a reference. All three *atxn1* genes were already maternally expressed at the single-cell stage (zygote). Differential expression levels of *atxn1a*, *atxn1b*, and *atxn1l* were mainly detected during the early embryonic stages. The expression of *atxn1l* increased slightly in the blastula period at 3 hpf (about 1000 cells) and remained at a constant level in all later stages and in the adult brain. The expression level of *atxn1a* in the zygote (single-cell stage) was maintained in the blastula period (3 hpf) and after the onset of gastrulation at 6 hpf (50% epiboly), but decreased by 12 hpf in the segmentation period with the lowest expression level at 1 dpf (pharyngula period). The expression of *atxn1a* increased again at 2 dpf (hatching period) and 3 dpf (protruding-mouth stage) and remained at a constant level during larval development from 4 dpf to 10 dpf and in the adult brain. In contrast to the *atxn1a* and *atxn1l* transcripts, the maternal component of the *atxn1b* transcripts was rapidly degraded, and no embryonic expression was detected in the blastula period (1000-cell stage). The RT-PCR analyses revealed minimal levels of *atxn1b* during the gastrula period at 6 hpf (shield stage), and noticable expression reappeared during the segmentation period at 12 hpf during times of early brain development and was consistently expressed at higher levels from 1 dpf to 10 dpf and in the adult brain. In summary, all *atxn1* genes were expressed at similar levels in the zygote, the larval stages, and the adult brain. However, the expression of *atxn1a* decreased during the second half of the first day of development while *atxn1b* decreased during early embryonic stages. *Atxn1b* reappeared as zygotic expression during the stages of onset of neuronal development while the expression of *atxn1l* remained constant during all embryonic and larval stages analyzed.

## 3. Discussion

In neurodegenerative diseases such as hereditary dominant ataxias, neuronal cells in the brain lose their function over time and finally die. To date, there is no cure for these movement disorders. There is a critical need to identify the pathological processes and to develop new approaches for treatment or prevention. Therefore, the zebrafish *Danio rerio* has already become an increasingly important model organism for human neurodegenerative diseases such as the autosomal dominant spinocerebellar ataxias SCA2, SCA3, SCA6, SCA8, and SCA18 (for review see [4]). It could be important to expand the range of zebrafish models also to SCA1, which is largely caused by a proteotoxic gain-of-function mechanism due to an expansion of a polyglutamine stretch in the human ATXN1 protein [5,17]. ATXN1 shows a nuclear–cytoplasmic localization, interacts with large protein complexes, binds RNA, and is thought to be involved in transcriptional repression [18]. Interestingly, although SCA1 is caused by an expansion of a CAG repeat in the coding region of the *ATXN1* gene, no repeats were found in the corresponding paralog gene region of rodents, prosimian, and New World monkeys. Perfect (uninterrupted) CAG repeats were found in Old World monkeys and interrupted CAG repeats in hominoids [19]. This suggests that the disrupted CAG structure of the *ATXN1* gene was acquired gradually during human evolution, and the interruption of the CAG sequence is associated with a slower progression of neurodegeneration.

Likewise, little is known about *atxn1* genes in zebrafish. Three *atxn1* genes are indicated in the Zebrafish Information Network Database (ZFIN, https://zfin.org/ (accessed on 3 March 2021)), two *ataxin-1* genes, annotated as *atxn1a* and *atxn1b* as well as one *ataxin-1-like* gene, *atxn1l*. In a recent work, three Atxn1 proteins, Atxn1a, Atxn1b, and Atxn1l have been described in zebrafish. Using a cross-specific anti-ATXN1 antibody that recognizes all three zebrafish proteins, it was shown that all are expressed in the cerebellar anlage of embryos and in the adult zebrafish cerebellum [16]. Three conserved regions of high homology were identified between the human ATXN1 and the three zebrafish proteins, including the N-terminus, the AXH domain as an important mediator of protein-protein interactions, and the C-terminus. The long polyglutamine repeat is not conserved in zebrafish [16]. Our comparative sequence alignment revealed that this is also the case in all other vertebrate species examined. The polyglutamine repeat is only found in humans and chimpanzees. We compared the protein sequence of human ATXN1 and ATXN1L and other vertebrate models to the zebrafish ortholog proteins in relation to the size, the similarity and the phylogenetic distance. Besides the species-specific polyglutamine stretch, the length of the human ATXN1 (815 amino acid residues, aa) is in the range of the other somewhat smaller ortholog proteins in chimpanzees, rats, mice, chickens, frogs and zebrafish (including the zebrafish Atxn1a (881 aa) and the longer zebrafish Atxn1b (828 aa)). A sequence comparison revealed 46% (Atxn1a) and 33% (Atxn1b) amino acid identities to ATXN1. The smaller ATXN1L protein (689 aa) and the vertebrate orthologs have similar sizes. The zebrafish Atxn1l protein (865 aa) has a comparably longer amino acid chain than the other selected vertebrate orthologs and is 42% identical to the human protein. These data were used to elucidate the phylogenetic relationship between the zebrafish proteins Atxn1a and Atxn1b as well as their human and other vertebrate orthologs. Our phylogenetic analysis indicated two clusters for human ATXN1 proteins and ATXN1L proteins in vertebrates. The ATXN1 cladogram revealed a close relationship in hominids and rodents but much less so in chickens, mice, and zebrafish. The same phylogenetic relationships were found for the ATXN1L cluster between species. The phylogenetic analysis of all three zebrafish proteins revealed Atxn1a as the evolutionarily closer protein variant to the human ATXN1 protein. In contrast to Atxn1a, we showed that the zebrafish variant Atxn1b is phylogenetically closer to its own Atxn1l paralog and that of other vertebrates.

The two Atxn1a and Atxn1b protein variants in zebrafish result from a duplication of the *atxn1* gene and have a common phylogenetic origin. The zebrafish belongs to the very diverse infraclass of *Teleostei* that accounts for about half of the living vertebrates and over 96% of all fish species [20,21]. The teleost lineage underwent a whole-genome duplication event (TGD) about 320 million years ago, resulting in two copies of each gene in the genome [22]. Such paralogous genes were presumably redundant, and one copy was lost randomly during further evolution [23]. This was likely the case for the *atxn1l* gene, with only one copy of the gene present on the zebrafish chromosome seven minus strand. The *atxn1a* gene maps on chromosome 19 plus strand and the *atxn1b* gene on chromosome 16 minus strand. These chromosomes were identified as paralogous chromosomes [24]. We performed a comparative synteny analysis of the human *ATXN1* gene locus to the zebrafish *atxn1a* and *atxn1b* genomic loci. We identified the *atxn1b* gene with conserved synteny to the human ortholog. No synteny was found for the zebrafish *atxn1a* locus. However, when both paralogs are maintained, accumulations of mutations can affect one gene copy differently to the other and lead to new functional properties or asymmetric acceleration of the evolutionary rate [25].

We analyzed and compared the genetic structures and expression patterns of the three *atxn1* genes and could identify similarities and differences in the zebrafish. The *atxn1a* gene spans 198 kb of genomic DNA and is organized in eight exons that are each employed to express four different transcript variants, which differ from one to three exon sequences in the 5′ untranslated region (5 ′ UTR), but all of them use exon E4 and E5, which contain the coding sequences. This is similar for the *atxn1b* gene, which spans 35 kb with five exons that transcribe two different variants, X1 and X2. Both differ in three different exons in the 5′ UTR (X1: E1′ and E2′; X2: E1) and use the larger exons E2 and E3 that harbor the coding sequences. This variability of the 5′ UTR is similar to the human ATXN1 gene, which is much larger (450 kb) and organized in nine exons. The first seven exons are located in the 5′ UTR, while exons E8 and E9 contain the coding region and the 3′ UTR [17]. We confirmed all in silico suggested *atxn1a* (X1–X4) 5′ UTR transcript variants and the *atxn1b* X1variant via RT-PCR and sequencing. However, in the case of *atxn1b* X2 variant, the predicted exon E2′ could not be confirmed experimentally. Sequencing revealed that this exon is either weakly expressed or not expressed and not amplifiable by RT- PCR or spliced out in the *atxn1b* X2 variant. Together, *atxn1a* and *atxn1b* variants contain variable noncoding exons in the 5′ UTR. It is likely that these noncoding exons undergo alternative splicing in different tissues of the zebrafish. This leads to the assumption that the transcriptional and translational regulation of *atxn1a* and *atxn1b* may be complex and tissue dependent in zebrafish. In contrast, the *atxn1l* gene in zebrafish contains only three exons that are distributed in a region over 18 kb of the genomic DNA and encode only one transcript. The predicted 5′ UTR exon sequence of *atxn1l* was also experimentally confirmed in our study.

Interestingly, the protein size of human ATXN1 (815 aa) with a molecular mass of 88 kD is almost the same to the gene products of Atxn1a (881 aa) and Atxn1b (828 aa). However, the human ATXN1 mRNA (NM_000332.3) is much longer (10,636 nt) than all the predicted zebrafish transcript (5–8 kb) variants. In humans, the last coding exon contains a 8288 bp long 3′ untranslated region (3′ UTR). In contrast, the predicted 3′ UTRs of *atxn1* transcripts in zebrafish are much shorter. In addition to the confirmation of the 5′ends of the *atxn1* variants in zebrafish, we could also experimentally confirm the coding regions and most of the predicted 3′ UTR by RT-PCR using primer pair combinations covering the complete predicted sequences. However, we failed to amplify the exact 3′ prime end in all three *atxn1* transcript variants. We performed several 3′ RACE (rapid amplification of cDNA ends) experiments using different sets of forward primers at the 3′ end of the cDNA and reverse primers containing oligo-(dT) universal amplification sequence and also prolonged extension times in PCR. We could not amplify any reliable cDNA amplicons in several attempts. We assume that the 3′ UTRs of *atxn1* transcripts are likely much longer or different than predicted and will need to be studied in the future.

In addition to the conserved organization of the *ATXN1* and *ATXN1L* genes between humans and zebrafish, we were interested in whether this was also true for their expression patterns. In human, ATXN1 expression domains were identified by immunoblot analysis in different brain regions, but also in nonneuronal tissues such as the liver, heart and skeletal muscle. In nonneuronal tissues, ATXN1 is predominantly located in the cytoplasm, in contrast to the brain where the neurons of the cortex, the basal ganglia, and the pons show a nuclear localization of ATXN1. A nuclear–cytoplasmic localization was shown for Purkinje cells of the cerebellum [18]. Purkinje cells are the key neurons in spinocerebellar ataxia type 1 because an expansion of the trinucleotide CAG repeat in the *ATXN1* gene leads to nuclear inclusions of the altered mutated protein in these neurons involved in the pathogenesis of SCA1 [5,17,26,27]. In zebrafish, Atxn1a and Atxn1b contain a nuclear localization signal (NLS) and both proteins localize to the nucleus in tissue culture experiments. Like in human, the zebrafish Atxn1l protein is mostly cytoplasmic, but when co-expressed in COS-1 cells, both are predominantly found in the nuclei [16]. This strengthens the previous assumption that ATXN1 may support the shuttling of ATXN1L to the nucleus and interact in large protein complexes to cause SCA1 neuropathology [28]. RT-PCR was used to demonstrate that each member of the *atxn1* gene family is expressed in the adult zebrafish cerebellum, and *atxn1a* and *atxn1b* are expressed in the cerebellar anlage of 28 hpf embryos, supporting the role of both genes in cerebellar development [16]. Our detailed expression analysis of all *atxn1* members in zebrafish comprehensively expands the expression data of *atxn1* in the embryos and the larvae of this model system. Our data show a spatiotemporal expression of all three *atxn1* family members with overlapping expression domains during early embryogenesis followed by different expression levels and domains during further development.

*Atxn1a* is uniformly expressed in the embryo during the segmentation phase with increasing levels in the diencephalon at 1 dpf. The expression domains extend to areas such as the epiphysis, the telencephalon, the metencephalon, the rhombencephalon, the optic tectum, and the cerebellum during further developmental stages until 4 dpf. *Atxn1a* expression persists in the gills but decreases or disappears almost completely in the rhombencephalon at the larval stages of 7 dpf and 10 dpf. At this stage, a rather low and diffuse expression is found in the cerebellum. Interestingly, the expression domain of *atxn1a* more closely resembles the pattern of a*txn1l* than that of *atxn1b*, with the exception that *atxn1l* expression is enhanced in the rostral and caudal region during the segmentation phase, as compared to a more uniform expression of *atxn1a*. Then the expression domain of *atxn1l* shifts to the anterior part at 1 dpf and includes the developing CNS with the tegmentum, the diencephalon and the mesencephalon, the cerebellum, the metencephalon, and the myelencephalon at 2 dpf. *Atxn1l* expression also becomes downregulated in almost all brain regions when transcripts are still detectable in the tectum and the cerebellum, but primarily in the regions of gill arches from stages 4 dpf to 10 dpf. The spatiotemporal expression domain of *atxn1b* differs to some extent from those seen for the other two gene family members. A strong expression is already obvious in the telencephalic region at 1 dpf and extends to the brain regions of the tectum opticum, the cerebellum, and the rhombencephalon at 4 dpf. In contrast to *atxn1a* and *atxn1l*, *atxn1b* expression decreases only in the telencephalon, but persists in the midbrain and hindbrain region from 7 dpf to 17 dpf. Cryosectioning of the stained embryos confirmed the expression domains in the thalamus, the tectum opticum, the retina, the pineal gland, the cerebellum, and the rhombencephalon.

The RT-PCR analysis confirmed the expression of all *atxn1* genes during embryonic and larval stages in zebrafish. While *atxn1l* is continuously and uniformly expressed from fertilization until adulthood, *atxn1a* expression decreases during stage of early brain development but subsequently recovers to previous levels at 2 dpf. Instead, maternal *atxn1b* expression rapidly declines during the first hours of development and reappears as zygotic expression at 12 hpf when early brain development occurs and lasts until the adult stages. Together with the spatiotemporal expression profile observed with the WISH analysis, these findings argue that *atxn1b* is more strictly reflecting neuronal development, differentiation, and homeostasis.

Taken together, the results of all whole-mount in situ hybridization experiments on *atxn1* gene expression indicate that *atxn1a*, *atxn1b*, and *atxn1l* are upregulated primarily during embryonic development of the zebrafish brain, but only *atxn1b* is continuously expressed at higher levels in the midbrain and hindbrain during further larval development and is strictly confined to the cerebellum. This highlights the need to perform further experiments with the goal of identifying the function of these proteins in the zebrafish model in the future.

## 4. Materials and Methods

### 4.1. Bioinformatics

The phylogenetic analysis with human, chimpanzee, rat, mouse, chicken, frog and zebrafish Atxn1 and Atxn1l protein sequences (Table S1) was conducted with the Unipro UGENE software analysis tool using the PHYLIP neighbor-joining method and the Jones–Taylor–Thornton distance matrix model. The clustalW alignments were also performed by the Unipro UGENE software analysis tool. The annotated *atxn1* and *atxn1l* transcript sequence variants were downloaded from the NCBI database server (Table S3). The reference RNA sequences (refseq_rna) were blasted and aligned to Refseq Genome database (refseq_genomes) of *Danio rerio* (taxid:8955) (Table S3). Gene synteny was analyzed by genomic data comparison by the use of the NCBI database server.

### 4.2. Ethics Statement

Zebrafish (*Danio rerio*) were maintained in our fish facility according to the local animal welfare standards (Tierschutzgesetz §11, Abs. 1, Nr. 1) and the European Union animal welfare guidelines and legal regulations (EU-Directive 201_63) under the supervision of the local representative (Ordnungsamt Braunschweig) of the animal welfare agency (Permit # AZ 32.5./325-1-5-6-1). Zebrafish lines were maintained under constant 14 h light and 10 h dark cycles at 28 °C. All animals were treated according to the standard protocols [29] and to the Declaration of Helsinki for animal use and care. Embryos and larvae were anesthetized and killed in ice water according to protocols approved by the Niedersächsisches Landesamt für Verbraucherschutz und Lebensmittelsicherheit (Permit # Az: 33.19-42502-05-15A526, 22 March 2015; Permit # Az: 33.19-42502-05-16A070, 19 October 2016;), using tricaine as anesthetic.

### 4.3. Embryo and Larvae Treatment

Male and female zebrafish from the *brass* and *casper* line were put into breeding tanks the day before pairing. Fertilized eggs were collected after mating in the morning and incubated in fish water in a humidified incubator at 28 °C. Unfertilized eggs and cell debris were removed, and the fish water was replaced by 30% Danieau’s medium in the evening. PTU was added after gastrulation (about 10 h post-fertilization) to suppress residual pigmentation in embryos of the *brass* line. Danieau’s medium with PTU was exchanged daily until embryos or larvae were harvested. Embryos and larvae from the transparent *casper* line were raised in 30% Danieau’s without PTU.

### 4.4. RNA Isolation, RT-PCR, Cloning, Probe Synthesis

Total RNA was isolated from an adult zebrafish using peqGOLD RNAPur (peqLab). An amount of 2 µg of total RNA was reverse transcribed with oligo (dT) primers, a dNTP mix, and SuperScript III Reverse Transcriptase (Thermo Fisher Scientific, Waltham, MA, USA) for 1 h at 50 °C. For cloning zebrafish constructs of *atxn1a, atxn1b* and *atxn1l*, partial cDNA sequences were amplified by using primers as indicated (Table S4). The RT-PCR products were isolated (QIAGEN Gel Extraction Kit) and the purified fragments were cloned into the pGEM-T Easy vector. Plasmids were linearized accordingly and transcribed using T7 or Sp6 RNA-polymerases to generate DIG-labeled antisense and sense riboprobes using the DIG RNA labeling mix (Roche, Basel, Switzerland). Synthesized probes were purified by the RNeasy kit (Qiagen, Hilden, Germany) and checked for quality after electrophoresis using a denaturing 1.2% agarose gel containing formaldehyde as described elsewhere [30]. For comparative examination of *atxn1a, atxn1b* and *atxn1l* transcript levels, RT-PCRs were performed with total RNAs isolated from whole-bodies of embryos and larvae at different stages of zebrafish development and from an adult brain. Primer pairs for *atxn1a*, *atxn1b*, and *atxn1l* were selected to obtain amplicons of approximately the same sizes as indicated (Table S5).

### 4.5. Whole-Mount In Situ Hybridizations

In situ hybridization was performed in embryos and larvae according to a recently published all-age whole-mount in situ hybridization protocol [30]. In brief, samples were stored in 100% methanol and rehydrated in a decreasing series of ethanol. Tissues were permeabilized by acetone–xylol and bleached in 6% H_2_O_2_ before prehybridization. Hybridization was performed with riboprobes (50 ng/mL) in hybridization solution at 65 °C for an extended period of 60 h. Following the washing steps, embryos and larvae were incubated in a dilution with anti-DIG antibodies (1:4000) over night, followed by extensive washings for three days. Staining was performed using BM purple (Roche) as substrate for about 28 h.

### 4.6. Microscope Equipment and Imaging

All stained embryos and larvae were stored in 70% ethanol–H_2_O until imaging. The samples were transferred onto a microscope slide and incubated for equilibration in 90% glycerol–H_2_O for a few minutes. The embryos and larvae were then positioned for the desired focus plane under a stereomicroscope (Leica MZFLIII, Leica Biosystems, Wetzlar, Germany). The visualized gene expression pattern of the tissues was recorded using the Nikon DS-Fi3 microscope camera system and the NIS-Elements D software.

## 5. Conclusions

In summary, human ATXN1 is a chromatin-binding protein and is involved in the pathogenesis of spinocerebellar ataxia 1 (SCA1) when extensions of polyglutamine residues result in a proteotoxic gain of function. ATXN1 and its paralog ATXN1L are also found in other vertebrates including the zebrafish, which became a prominent model system for human neuronal disorders. We analyzed the phylogenetic relationship of recently identified zebrafish Atxn1a, Atxn1b, and Atxn1l proteins among the vertebrates. We identified zebrafish Atxn1a with the closest relation to other protein members of vertebrates, followed by Atxn1b and Atxn1l. The zebrafish genes are located on different chromosomes and show a variability of exon usage in the 5′ UTR of the predicted transcripts. We could confirm four transcript variants for *atxn1a*, two for *atxn1b,* and one for *atxn1l* by RT-PCR, and we analyzed the expression pattern of each gene during embryonic and larval development. All three family members are expressed in brain regions including the cerebellar anlage during early embryogenesis and might play a crucial role in the development of the cerebellum. Interestingly, as found before in humans, a*txn1a* and *atxn1l* also have similar spatiotemporal expression domains in zebrafish. All three genes are maternally expressed, yet while *atxn1b* expression vanishes quickly and reappears during the onset of brain development, *atxn1a* expression is transiently downregulated during the stages of early brain development. *Atxn1b* is strictly expressed in the cerebellum during all stages of brain development and persists until adulthood. This knowledge of gene structures and differential gene expression of *atxn1* gene family members is a beneficial prerequisite for further investigations regarding SCA1 pathogenesis in the zebrafish model.

## Data Availability

Data and tools described in this manuscript are available upon request.

## Supplementary Materials

The following are available online at https://www.mdpi.com/article/10.3390/ijms222111348/s1.
