# Supplementary material for: Structural Analysis and Spatiotemporal Expression of Atxn1 Genes in Zebrafish Embryos and Larvae
Source: Int J Mol Sci. 2021 Oct 21;22(21):11348. doi: 10.3390/ijms222111348 (PMC8583371; doi:10.3390/ijms222111348)
Supplement: Supplementary file 1 [file ijms-22-11348-s001.zip › ijms-1312707-supplementary.pdf]

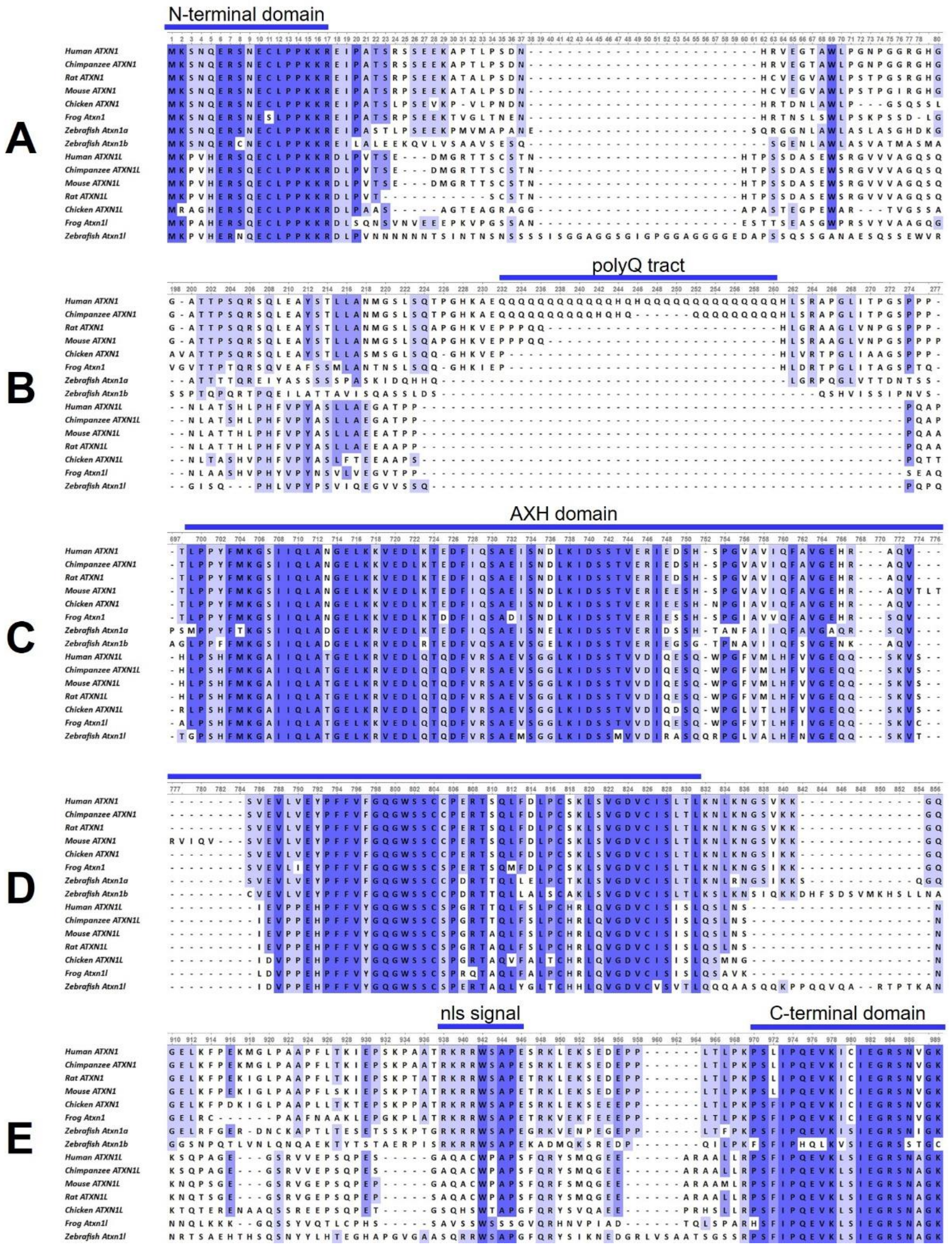

Figure S1. ATXN1 and ATXN1L proteins are highly conserved during vertebrate evolution. ClustalW alignment was performed using the Unipro UGENE software analysis tool. The following protein sequences were used: ATXN1 paralogs (*Homo sapiens*: NP_001121636.1; *Pan troglodytes*: XP_001170170.2; *Rattus norvegicus*: XP_038951297.1; *Mus musculus*: NP_001186234. 1; *Gallus gallus*: XP_003640803.1; *Xenopus tropicalis*: XP_002932738.1; *Danio rerio*: NP_001038291.1; *Danio rerio*: XP_005158216. 1) and ATXN1L paralogs (*Homo sapiens*: NP_001131147.1; *Pan troglodytes*: XP_016785624.1; *Mus musculus*: NP_001074399.1; *Rattus norvegicus: XP_008770785.2; Gallus gallus: XP_015148186.1; Xenopus tropicalis: XP_002937622.1; Danio rerio: XP_001333781.2*). Domains with high homology or specificity are marked: (**A**) N-terminal domain; (**B**) polyglutamine tract; (**C** and **D**) AXH domain; (**E**) nuclear localization signal, nls; C-terminal domain.

Table S1. Ataxin-1 proteins in vertebrates.

Vertebrate species	Accession number	Protein	Amino acid residues
Homo sapiens	NP_001121636.1	Human ATXN1	815 aa
Pan troglodytes	XP_001170170.2	Chimpanzee ATXN1	809 aa
Rattus norvegicus	XP_038951297.1	Rat ATXN1	789 aa
Mus musculus	NP_001186234.1	Mouse ATXN1	799 aa
Gallus gallus	XP_003640803.1	Chicken ATXN1	798 aa
Xenopus tropicalis	XP_002932738.1	Frog Atxn1	792 aa
Danio rerio	NP_001038291.1	Zebrafish Atxn1a	781 aa
Danio rerio	XP_005158216.1	Zebrafish Atxn1b	827 aa
Homo sapiens	NP_001131147.1	Human ATXN1L	689 aa
Pan troglodytes	XP_016785624.1	Chimpanzee ATXN1L	689 aa
Mus musculus	NP_001074399.1	Mouse ATXN1L	687 aa
Rattus norvegicus	XP_008770785.2	Rat ATXN1L	679 aa
Gallus gallus	XP_015148186.1	Chicken ATXN1L	690 aa
Xenopus tropicalis	XP_002937622.1	Frog Atxn1l	698 aa
Danio rerio	XP_001333781.2	Zebrafish Atxn1l	765 aa

Table S2. Gene structure and exon usage of predicted transcripts in *atxn1* genes in zebrafish.

A

atxn1a gene structure											
**X1 variant**			**X2 variant**			**X3 variant**			**X4 variant**		
	Exons (bp)	Introns (bp)		Exons (bp)	Introns (bp)		Exons (bp)	Introns (bp)		Exons (bp)	Introns (bp)
E1	180		E1´	20							
I1		55036	I1´		4164						
E2	189		E2	189					E2´	95	
I2		79922	I2		79922				I2´		81
E3	101		E3	101		E3´	408		E3	101	
I3		54652	I3		54652	I3´		10480	I3		54652
E4	2097		E4	2097		E4	2097		E4	2097	
I4		2277	I4		2277	I4		2277	I4		2277
E5	3361		E5	3361		E5	3361		E5	3361	
all	**5928**	**191887**	all	**5768**	**141015**	all	**5866**	**12757**	all	**5654**	**57010**
**X1 gene region 197815 bp**			**X2 gene region 146783 bp**			**X3 gene region 18623 bp**			**X4 gene region 62664 bp**		

B

atxn1b gene structure					
**X1 variant**			**X2 variant**		
	Exons (bp)	Introns (bp)		Exons (bp)	Introns (bp)
			E1´	1079	
			I1´		17009
E1	351		E2´	58	
I1		4414	I2´		11528
E2	2091		E2	2091	
I2		93	I2		93
E3	3943		E3	3943	
all	**6385**	**4507**	all	**7171**	**28.630**
**X1 gene region 10892 bp**			**X2 gene region 35801 bp**		

C

atxn1l gene structure		
**1 variant**		
	Exons (bp)	Introns (bp)
E1	185	
I1		5427
E2	1112	
I2		6075
E3	4923	
all	**6220**	**11502**
**gene region 17722 bp**		

Table S3. Zebrafish *atxn1a*, *atxn1b* and *atxn1l* transcript variants and primer sequences used for RT-PCR.

Transcript variants	Accession number	Upper/Lower Primer	Primer sequence
atxn1a X1	XM_017352239.2	UX1a<br>LX1-4a	5´-ACACACACACTCTCTCACTGGAGG-3<br>5´-TGTCCTGTGCCTATGGTCGGATTG-3´
atxn1a X2	XM_009294318.3	UX2a<br>LX1-4a	5´-GACAAGGAAGGGTTTTTGTGGAATGGT-3´<br>5´-TGTCCTGTGCCTATGGTCGGATTGG-3´
atxn1a X3	XM_005159620.4	UX3a<br>LX1-4a	5´-GGGCGCTCTGCCAGCTG-3´<br>5´-TGTCCTGTGCCTATGGTCGGATTG-3´
atxn1a X4	XM_005159619.4	UX4a<br>LX1-4a	5´-CACTAGTATGAGTGGAGTAAAAGCACCCG-3´<br>5´-TGTCCTGTGCCTATGGTCGGATTG-3´
atxn1b X1	XM_005158159.4	UX1b<br>LX1-2b2	5´-GTCGAAGGAAGTGGAGGAGAGTGAAG-3´<br>5´-TGGGAGTCTGGCTGCTTTGC-3´
atxn1b X2	XM_689102.6	UX2b<br>LX1-2b1	5´-CATCGTTTTGCAGTTCTGTGGTGAATGAG-3´<br>5´-TTGCTATGTGATGGGCCTGCATTG-3´
atxn1l	XM_001333745.7	U1l<br>L1l	5´-GATCAGCGGCTCCTACCTGACG-3´<br>5´-GCACTTGCGTTGCAGCTTGCTC-3´

Table S4. Zebrafish *atxn1a*, *atxn1b* and *atxn1l* transcripts and primer sequences used for cloning of templates for probe synthesis.

Transcript	Accession number	Upper/Lower Primer	Primer sequence	Amplified region	Construct length
atxn1a X1	XM_017352239.2	UX1a2<br>LX1a2	5´-CTGTCTCCCGCAGTTCCTCCTTATC -3´<br>5´- GCCCTGGCCGAACACAAAGAAAG-3´	1861-2618	758 nt
atxn1b X1	XM_005158159.4	UX1b2<br>LX1b2	5´-CCAACAATGCAGGCCCATCACATAG-3´<br>5´-CAGACATCGCCCACAGAGAGTTTG-3´	722 – 2570	1849 nt
atxn1l	XM_001333745.7	U1l2<br>L1l2	5´-GCTAGAAGTGTGCGGCTGATGC-3´<br>5´-GAGAAGAGGCAGAGAGAGGACGAG-3´	1172 – 3219	2048 nt

Table S5. Zebrafish *atxn1a*, *atxn1b, atxn1l* and *β-actin* transcripts and primer sequences used for RT-PCR.

Transcript	Accession number	Upper/Lower Primer	Primer sequence	Amplified region	Amplicon length
atxn1a X1	XM_017352239.2	UX1a2<br>LX1a2	5´-CTGTCTCCCGCAGTTCCTCCTTATC -3´<br>5´- GCCCTGGCCGAACACAAAGAAAG-3´	1861 - 2618	758 bp
atxn1b X1	XM_005158159.4	UX1b2<br>LX1b3	5´-CCAACAATGCAGGCCCATCACATAG-3´<br>5´-TGGGAGTCTGGCTGCTTTGC-3´	722 - 1334	613 bp
atxn1l	XM_001333745.7	U1l3<br>L1l3	5´-GAGTGCCTCCCTCCCAAGAAGC-3´<br>5´-GCACTTGCGTTGCAGCTTGCTC-3´	323 - 989	667 bp
β–actin	NM_131031.2	U1<br>L1	5´-TCCCCTTGTTCACAATAACC-3´<br>5´-TCTGTTGGCTTTGGGATTC-3´	67 - 449	383 bp
